# Design, Implementation, and Evaluation of a Head and Neck MRI RF Array Integrated with a 511 keV Transmission Source for Attenuation Correction in PET/MR

**DOI:** 10.3390/s19153297

**Published:** 2019-07-26

**Authors:** Lucia Isabel Navarro de Lara, Roberta Frass-Kriegl, Andreas Renner, Jürgen Sieg, Michael Pichler, Thomas Bogner, Ewald Moser, Thomas Beyer, Wolfgang Birkfellner, Michael Figl, Elmar Laistler

**Affiliations:** 1 Center for Medical Physics and Biomedical Engineering, Medical University of Vienna, Waehringer Guertel 18-20, 1090 Vienna, Austria; 2 Institute of Applied Physics, Vienna University of Technology, Wiedner Hauptstrasse 8-10/134, 1040 Vienna, Austria

**Keywords:** PET/MR, attenuation correction, radiofrequency coil, receive-only array, 3 Tesla

## Abstract

The goal of this work is to further improve positron emission tomography (PET) attenuation correction and magnetic resonance (MR) sensitivity for head and neck applications of PET/MR. A dedicated 24-channel receive-only array, fully-integrated with a hydraulic system to move a transmission source helically around the patient and radiofrequency (RF) coil array, is designed, implemented, and evaluated. The device enables the calculation of attenuation coefficients from PET measurements at 511 keV including the RF coil and the particular patient. The RF coil design is PET-optimized by minimizing photon attenuation from coil components and housing. The functionality of the presented device is successfully demonstrated by calculating the attenuation map of a water bottle based on PET transmission measurements; results are in excellent agreement with reference values. It is shown that the device itself has marginal influence on the static magnetic field B_0_ and the radiofrequency transmit field B_1_ of the 3T PET/MR system. Furthermore, the developed RF array is shown to outperform a standard commercial 16-channel head and neck coil in terms of signal-to-noise ratio (SNR) and parallel imaging performance. In conclusion, the presented hardware enables accurate calculation of attenuation maps for PET/MR systems while improving the SNR of corresponding MR images in a single device without degrading the B_0_ and B_1_ homogeneity of the scanner.

## 1. Introduction

Multi-modality imaging provides valuable information for patient diagnosis. Specifically, the combination of positron emission tomography (PET) with magnetic resonance imaging (MRI) is very promising [1,2,3], as MRI examinations are highly complementary to PET, offering a wide range of high-contrast imaging sequences without employing additional ionizing radiation. However, the combination of PET and MRI represents a major challenge for molecular imaging technology, particularly regarding the development of PET-compatible radiofrequency (RF) coils for MR signal detection, and the implementation of novel attenuation correction (AC) methods, which do not rely on the availability of computed tomography (CT)-like transmission information.

From an MR perspective, radiofrequency (RF) coil arrays placed as close as possible to the region of interest can be used to increase image sensitivity and acquisition speed compared to single-channel coils [4,5]. In PET/MR systems, the RF coils are located within the PET field-of-view (FOV), and thus, may degrade PET image quality due to additional attenuation and scatter. Therefore, the design and development of RF coil arrays for integrated PET/MR systems poses new challenges and requirements compared to MR-only applications. Photon attenuation due to the RF coil itself has to be minimized, and the remaining effect should be characterized precisely so as to enable efficient data correction [6,7].

Attenuation correction is crucial for high-quality PET acquisitions. Standard methods for MR-based attenuation correction (MR-AC) [8] rely on the retrospective segmentation of MR images acquired with standardized MR sequences. Commercially available PET/MR systems offer fast MR sequences dedicated to deriving MR-based attenuation correction factors (ACF) employing either Dixon water/fat separation technique-based MR acquisitions [9,10,11,12,13], T_1_-weighted MR images segmented into air, lungs, and soft tissue, or a combination of an atlas-based approach and segmentation of T_1_-weighted images for deriving ACF. All standard methods have in common that bone attenuation is neglected to a significant extent, and; therefore, standard MR-AC leads to a systematic underestimation of the reconstructed activity distribution, particularly relevant in head and neck applications. In essence, none of the standard MR-AC methods today fulfill all of the requirements for an ideal attenuation correction method for PET/MR [14]. In order to overcome the limitations of standard MR-AC, alternative methods for AC have been proposed [15,16]. However, none of them are currently implemented in routine PET/MR systems. Template- and atlas-based methods support the estimation of areas containing bone using adaptive shape models or similar shape priors from previous segmentations, also from CT [8,17,18,19,20]. Ultra-short echo time (UTE) sequences allow for the measurement of bony structures; however, the method has not yet been demonstrated to work reproducibly well in situations with complex anatomy [12,21]. Known issues are air-tissue interfaces, for instance in the frontal sinuses.

Approximately 20 years ago, Jones et al. [22,23] presented a setup for attenuation measurement in PET-only systems called the “liquid drive”. It consisted of a hose and a pump driving a radioactive point source in a tube on a helical path and was installed in a single prototype PET system. However, the advent of PET/CT rendered such attenuation measurement technologies obsolete. Nonetheless, an effort that aims in this direction—the use of ^18^F as an external transmission source in a cylindrical structure—was presented by Mollet et al. [24,25]. In this case; however, the photons emitted by the external source were identified by a time-of-flight (TOF) measurement; thus, this approach is limited to PET systems with TOF information available.

The goal of our work is to adopt the “liquid drive” approach for non-TOF PET/MR imaging by adding information on the spatio-temporal localization of the external transmission source, and by fully integrating such a device within a highly sensitive MR-receiver array; thus, allowing for accurate AC mapping directly at the appropriate energy of 511 keV. The developed setup for attenuation correction tailored to meet the needs of clinical PET/MR comprises a transmission source (TxS) system that moves a point-like source on a helical path around the patient’s head and an integrated head and neck MR coil, designed for a 3T PET/MR system [26].

Here, we present the design and development of the MR receive array that is fully integrated with the TxS system. We describe how the RF coil array is designed in a way that PET attenuation by the coil itself is minimized, and how the impact of the TxS system on the MR coil performance was investigated. Further, an evaluation of the developed RF array in terms of sensitivity and parallel imaging performance is presented, as well as a comparison to a commercial state-of-the-art coil array for head and neck PET/MR.

## 2. Materials and Methods

### 2.1. System Overview

The developed setup for accurate PET/MR attenuation correction consists of a hydraulic system for driving a transmission source on a helical path around the patient’s head (hereafter referred to as TxS system) and a head and neck MR receive-only coil array. These two parts are fully integrated within one device. AC maps are calculated from the ratio of a blank scan and a transmission scan. The blank scan is acquired once a day, while the transmission scan is acquired simultaneously with the PET emission measurement for each patient individually.

**Figure 1:**
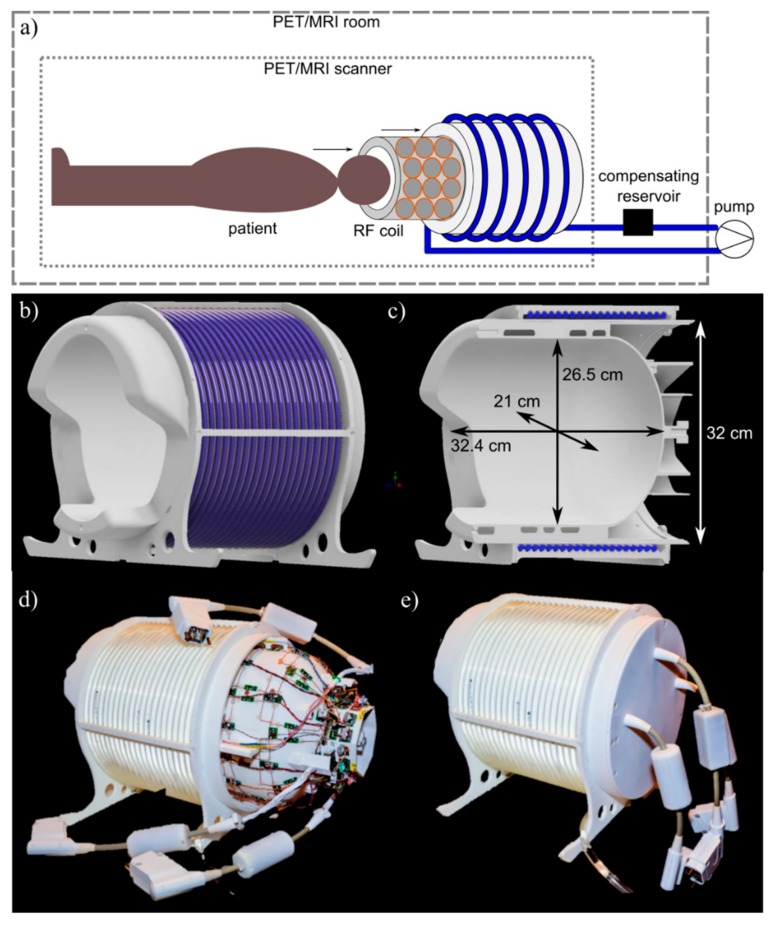
System overview. (**a**) Sketch of the complete setup consisting of transmission source (TxS) system and integrated radiofrequency (RF) receiver array, and its positioning relative to the PET/MR scanner. (**b**,**c**) 3D CAD drawing of the mechanical support with mounted polyurethane hose. The coil housing is shown in white, and the hose in blue. (**d**) Photograph of the complete RF coil integrated with the TxS system, with RF coil slid slightly out of the surrounding TxS system, and (**e**) in final, mounted configuration.

An overview of the complete setup is shown in Figure 1a. The hydraulic system consists of a polyurethane hose connected to a centrifugal pump. The hose is wound around the outer shell of the RF coil following a helical shape covering the complete PET field-of-view (FOV). The hose is filled with purified heavy water (D_2_O) to avoid contaminating ^1^H-MR signal. The transmission source is a refillable pellet made of polyethylene with a holding capacity of 0.4 mL. Information about the position of the pellet during scanning is extracted from the PET list-mode data. This allows for separation of emission and transmission data during reconstruction. The overall weight of the developed system in its final configuration including RF coil, housing, hose, and 1 L of D_2_O is 6.6 kg.

The developed hardware was designed and implemented on a Biograph mMR system (Siemens Healthcare, Erlangen, Germany). This PET/MR system consists of a combination of a state-of-the-art wide-bore 3T MR scanner with a fully integrated PET detector. Typically, the integrated body coil of the system is used as the RF transmit coil, as it is done in this study. The standard head receive coil provided with this system is a 16-channel head and neck coil (Siemens Healthcare, Erlangen, Germany). This coil is used as a reference coil for performance estimation of the coil developed in the presented work.

### 2.2. Mechanical Support and Coil Housing

A dedicated mechanical support (Figure 1b,c) was designed comprising an outer former to which the polyurethane hose is attached with fixation rods, and a slide-in inner former serving as RF coil holder and interface to the patient. The outer former has a circular cross-section to ensure a constant curvature for the hose of the liquid drive, while the inner former has an elliptical cross-section to match the shape of a human head more closely; further, the formers were designed with an opening for the shoulders of the scanned patient. At the rear end, the housing is covered by a plate with three cable outlets for the system cables connecting the RF coil to the PET/MR system. The complete housing rests on two struts shaped to accurately fit into the patient table of the scanner. The housing thickness was minimized to limit PET attenuation due to housing material, while maintaining mechanical stability. The overall maximum used wall thickness is 4 mm, with most areas of the housing being closer to 2 mm. All sub-parts of the mechanical support were designed using 3D CAD software and 3D-printed from laser-sintered polyamide.

### 2.3. RF Coil Design and Construction

**Figure 2:**
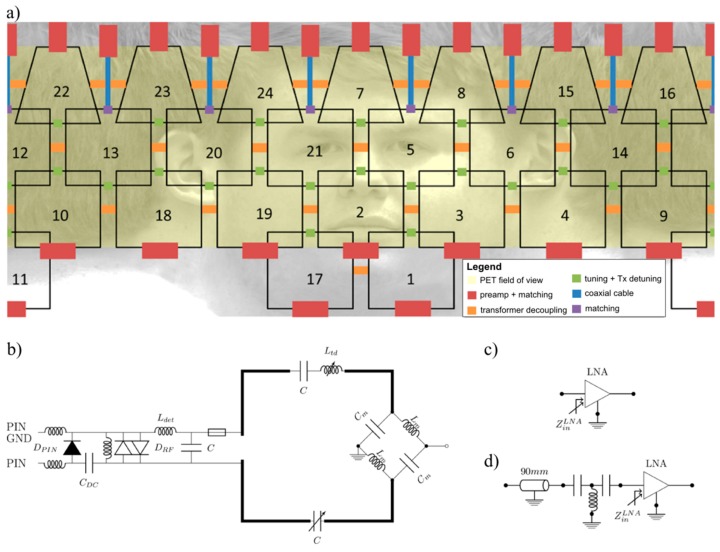
RF coil layout. (**a**) Unrolled layout of the 24-channel RF coil array; a description of the building blocks is given in the legend. Note the placement of heavily attenuating RF preamplifiers outside the PET field-of-view (FOV) (yellow area). (**b**) Circuit diagram of a single element. Two types of interfaces were used: (**c**) with direct connection to the preamplifier, and (**d**) with a coaxial cable, a phase shifter, and then the preamplifier.

The receive-only coil array, fully integrated with the TxS system, was designed for head and neck imaging applications in PET/MR. The number of channels for the array was chosen to represent a compromise between optimal coil size for the desired penetration depth (about 10 cm), geometrical arrangement, practical number of channels available per coil plug (eight channels per plug) and coverage of the head and neck, also allowing free space for the shoulders. The result of the design process was a 24-channel coil array consisting of four rows with 3/7/7/7 elements, respectively (Figure 2a). The elements of the first three rows are quadratic (9.5 cm × 9.5 cm), and the top row consists of seven trapezoidal elements (9 cm/5 cm × 10.5 cm).

The equivalent circuit of a single array element is depicted in Figure 2b. Each element was segmented into four parts by capacitors, and matched to 50 Ω using balun (balanced-to-unbalanced) matching networks [27] in order to minimize common mode currents. Within rows, elements were decoupled by transformer decoupling [28] with inductor L_td._, while for neighboring rows the overlapping method was employed. Transformer decoupling within rows was implemented to facilitate an even distribution of the square elements around the head, which is harder to achieve when overlap decoupling is used. Additionally, all elements were connected to low noise preamplifiers (HiQA, Carleton Place, ON, Canada) in a way to suppress current flow in each coil element and thereby reduce mutual coupling [29]. To ensure effective isolation of the constructed receive array from the transmit coil, active and passive detuning networks were implemented for each element. For both circuits, inductors L_det_ were manually wound from insulated copper wire. For active detuning, a PIN diode D_PIN_ was used (TEMEX, DH 80106, Pessac, France). This diode is biased through an RF choke (Coilcraft, 1812 CS, Cumbermauld, UK). The passive detuning circuits consist of a DC block capacitor C_DC_ (TEMEX, RF power capacitors, CPX, Pessac, France), RF fast diodes D_RF_ (Microsemi, UM9989, Aliso Viejo, CA, USA), and an RF choke inductor to short the switching currents. In addition, a series fuse of 315 mA nominal current rating was included in the circuit for each element as a safety measure in case the detuning circuits should fail.

During the whole coil design workflow, care was taken to minimize PET attenuation and scatter due to the presence of the MR receive array. As PET attenuation and scatter is related to electron density, metal in the PET FOV should be avoided. To this end, the basic coil elements were constructed using thin (0.75 mm diameter) copper wire, whereas inductors for decoupling and detuning circuits were wound from 0.5 mm diameter wire. Further, the preamplifiers were located outside the PET FOV [6]. For the first, second, and fourth row of coil elements, preamplifiers were located directly after the matching network, achieving efficient preamplifier decoupling by varying the input impedance of the preamplifier (Figure 2c). For the third row, a thin coaxial cable and a lumped element phase shifter were placed between matching network and preamplifier, to achieve preamplifier decoupling while placing these components outside the PET FOV (Figure 2d). The placement of all components is illustrated in Figure 2a, showing an unrolled view of the MR receive array with the PET FOV highlighted in yellow.

### 2.4. Monte-Carlo Simulations

**Figure 3:**
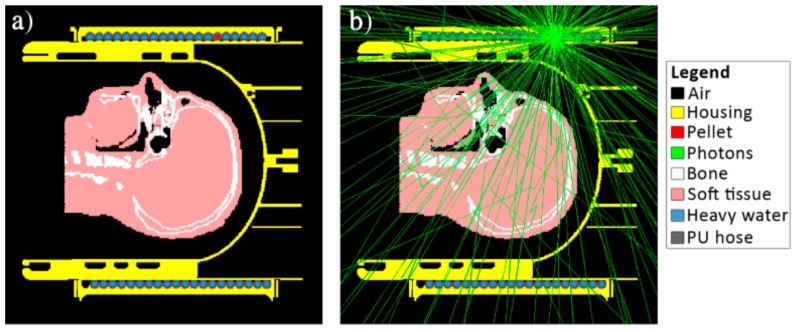
Monte-Carlo simulation of the developed system with a simplified head phantom. Tissue types: Air, bone, and soft tissue. (**a**) Setup without radiation; (**b**) Setup with tracks of the 511 keV annihilation photons (green lines). The coil elements are not visible in the sagittal cuts, as the diameter of these structures is below pixel size.

In order to assess the PET functionality of the proposed hardware, despite the loss in PET sensitivity due to the additional hydraulic system and the MR receiver coil, Monte-Carlo simulations were performed using the Geant4 Application for Tomographic Emission (GATE) [30]. To determine the global scatter-fraction, a virtual head phantom (Duke, Virtual Population, IT’IS Foundation, CH) simplified by assigning three different PET-relevant materials (bone, soft tissue, and air) to the different tissue types in the head, was placed inside the RF coil 3D CAD model (Figure 3a). The hardware model consisted of the housing, RF coil components, wires, and the liquid drive. A 20 MBq pellet running through the liquid drive was simulated as a point source. For the calculation of photon count loss, a virtual cylindrical water phantom (height: 140 mm, diameter: 130 mm) filled with 14.8 MBq of fluorine-18 with a simulation time of one second was used.

### 2.5. Bench Evaluation of the MR Receiver Array

Bench measurements were performed in order to adjust all components of the RF coil (i.e., tuning, matching, mutual and transmission decoupling), as well as to investigate the influence of the TxS system on the characteristics of the MR coil.

Tuning and matching of the array elements was achieved by measuring the reflection coefficient S_ii_ for each channel while the other elements were terminated with 50 Ω. Scattering parameters (S-parameters) were measured using a vector network analyzer (EB071C, Agilent, Santa Clara, CA, USA). A custom-made test rig was used to provide the required 10 V power supply for the preamplifiers and enable manual switching of the bias lines from forward (100 mA) to reverse bias (−30 V) for testing the active detuning circuit. Preamplifier decoupling was tested with a double-loop probe according to the method proposed by Reykowski et al. [29]. The measurements were performed after loading the RF coil with an in-house built, head-shaped gel phantom (6.3 L, 1% Agarose, 2 µmol/kg Gadolinium, 0.1% NaCl, and 0.05% NaN_3_), representing a load similar to a human head and T_1_ relaxation time of brain tissue (*T*_1,phantom_ = 2085 ms).

All RF coil components were adjusted with the liquid drive present. However, to evaluate its influence, resonance frequencies, and quality factors were also measured with the RF coil array alone.

### 2.6. MRI Evaluation of the Developed Hardware

To evaluate the usability of the developed hardware for MRI, the influence of the complete system on the RF transmit field generated by the scanner’s body coil was investigated in terms of flip angle maps. A 2D saturated Turbo FLASH [31] sequence (*T_R_*/*T_E_* = 6460 ms/1.91 ms, 6 slices, 5 mm slice thickness, FOV = 400 mm × 400 mm, MA = 224 × 224, and BW = 485 Hz/pixel, 80° nominal flip angle, 8° imaging flip angle, applying the saturation recovery module [31] to allow for a *T_R_* < 5 T_1_) was used to acquire these maps with phantom loading, once with the system present, and once without. For direct comparison, a ratio map of the flip angle distributions in both configurations was calculated.

Further, the impact of the developed system on the static magnetic field B_0_ was investigated by comparing maps of frequency deviations acquired with the body coil once with the system present, and once without, also probing two different shim configurations, i.e., the tune-up shim and the automatic shimming procedure implemented on the scanner. For this purpose, 3D multi-echo (*N_E_* = 3) gradient echo scans were acquired with a matrix size of 96 × 96 × 96 (voxels of 2.7 mm side length, 3 mm slice thickness), monopolar readout, *T_E_* = [5, 10, 17] ms, and *T_R_* = 21 ms; acquisition time was 3:13 min. Data were reconstructed using ASPIRE [32], and phase unwrapping was performed employing the UMPIRE method [33]. The B_0_ inhomogeneity was evaluated in terms of the standard deviation of frequency values calculated for both, the entire phantom, and a coronal slice.

The performance of the developed RF coil array was compared to that of the standard 16-channel Siemens head and neck coil delivered with the PET/MR system in terms of SNR. To this end, the pseudo-multiple replica method [34] was used on noise covariance and imaging data to calculate SNR maps for both head coils. 2D gradient-echo (GRE) images of the phantom were acquired (*T_R_*/*T_E_* = 470 ms/2.86 ms, flip angle 20°, 10 slices, 5 mm slice thickness, FOV = 349 mm × 349 mm, MA = 192 × 192, and BW = 322 Hz/pixel). To obtain noise covariance information [35], a sequence without RF excitation and gradients was used. The acquired raw data were processed by an in-house MATLAB script. The obtained SNR maps from both coils were co-registered using SPM12 for direct comparison.

For the assessment of the parallel imaging performance of the developed RF coil array, g-factor maps were calculated using the same data as for the SNR maps. The original raw data were decimated with acceleration factors *R* = 2, *R* = 3, and *R* = 4 in phase encoding direction. Then, applying again the pseudo-multiple replica and the GRAPPA reconstruction algorithm [36], g-factor maps were calculated using in-house written MATLAB scripts. The obtained g-maps were smoothed with a 3 × 3-pixel wide mean filter.

The developed coil was also tested in vivo on a healthy volunteer (male, 35 years) after written informed consent and following the guidelines of the Declaration of Helsinki to assess the achievable coverage of head and neck. For this purpose, an MPRAGE sequence [37] with the following parameters was used: *T_R_*/*T_E_*/*T_I_* = 2100 ms/2.44 ms/900 ms, GRAPPA 2, flip angle 9°, 160 slices, 1 mm slice thickness, FOV = 265 mm × 283 mm, MA = 230 × 256, and BW = 238 Hz/pixel.

### 2.7. PET Attenuation Coefficient Computation

To demonstrate the applicability of the developed device for PET attenuation correction measurements, the attenuation map of a 1 L water bottle without activity was calculated based on data from a 3-min transmission scan and a 17-min blank scan. Data were acquired using the pellet filled with 0.3 mL of ^18^F-FDG (activity 14 MBq) circulating through the hydraulic system. The 3D PET data were converted to 2D data sets using the single-slice rebinning algorithm [38]. Reconstruction of the ratio of blank and transmission scan was done by filtered back projection.

In clinical routine the workflow would consist of a blank scan in the morning. The duration of the blank scan is typically longer (~30 min) to achieve better counting statistics and, thus, less noise in the reconstructed AC map. A transmission scan (few min) would be acquired simultaneous to the PET emission scan for optimal patient throughput. A detailed evaluation of the AC mapping capability of the developed device including phantoms with emission activity, as well as a characterization of the liquid drive system and a detailed description of the reconstruction of the AC map can be found in the publication by Renner et al. [26]. In this evaluation study, the phantom was filled with water and included a Teflon cylinder and cylindrical air cavities with different diameters. Homogeneous regions in the phantom were successfully reproduced and air, water, and Teflon were clearly distinguishable. The reconstructed linear attenuation coefficient of water 0.096 ± 0.005 cm^−1^ is in full accordance with literature values. The smallest air cavities that could be recovered in the attenuation map had a diameter of 9.5 mm.

## 3. Results

### 3.1. Monte-Carlo Simulations

The simulated loss in photon counts due to the different parts of the system was 0.7% for the coil elements, 13% for the RF coil only, and 26% for the whole head and neck coil setup including the TxS system. The total global scatter-fraction using the head phantom was 16.4%.

### 3.2. S-Parameters, Q-Factors, Noise Correlation

**Figure 4:**
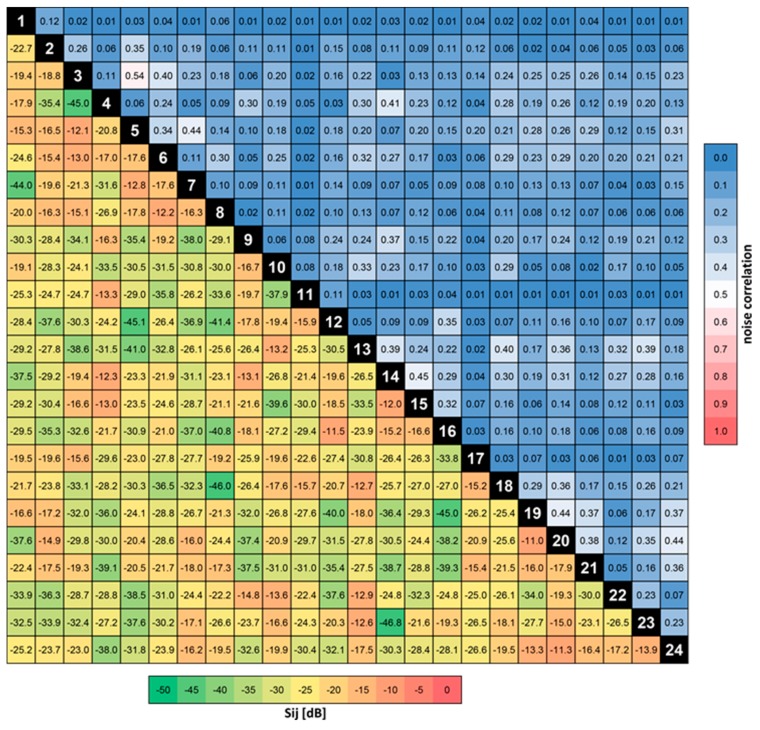
Radio frequency scattering parameters and noise correlation matrix. The lower triangle of the matrix shows the S-parameters, the upper triangle contains the noise correlation values between channels. In both cases the RF coil was loaded with the head-like gel phantom.

Matching of the individual array elements quantified in terms of the reflection coefficient was below −9 dB with an average of −11.6 dB. Figure 4 depicts the S-parameter and noise correlation matrices, showing inter-element coupling below −11 dB with a mean of −22.4 dB, and noise correlation below 0.54 with a mean value of 0.14.

Mean quality factors Q of 85 and 70 (ratio 1.2) for the unloaded and loaded coil elements, respectively, indicate that the total noise is dominated by coil noise. This is partly related to the choice of very thin wire for forming the elements in order to minimize PET attenuation, which was the major goal in the coil design process. Furthermore, some of the anterior elements are rather far away from the head—and, thus, only weakly loaded—since the coil could not be designed with a split top/bottom part housing due to the presence of the liquid drive.

The influence of the liquid drive system on the bench characteristics of the array was found to be negligible with a mean frequency shift of 0.1 MHz and a mean change of 2% for the unloaded Q-factor.

### 3.3. Influence on Static Magnetic Field (B_0_) and RF Transmit field (B_1_^+^)

**Figure 5:**
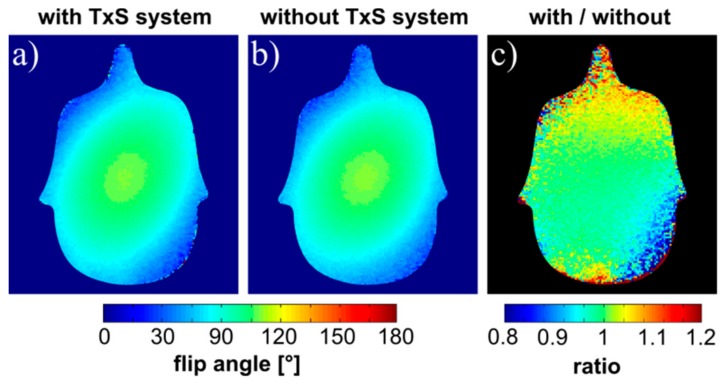
Flip angle maps. Maps represent the distribution of the RF transmit field generated by the body coil, which was also used for reception in these measurements. FA maps with (**a**) and without (**b**) the TxS system, (**c**) ratio map.

The relative deviation in flip angle (FA) maps acquired with the body coil with and without the receive array present was in general below 10%, with single pixels showing up to 15% deviation (see Figure 5). These maps demonstrate that the complete TxS system has marginal influence on the RF transmit field, and that the receive-only array is well isolated from the transmit coil, which ensures patient safety and avoids transmit field distortion.

**Figure 6:**
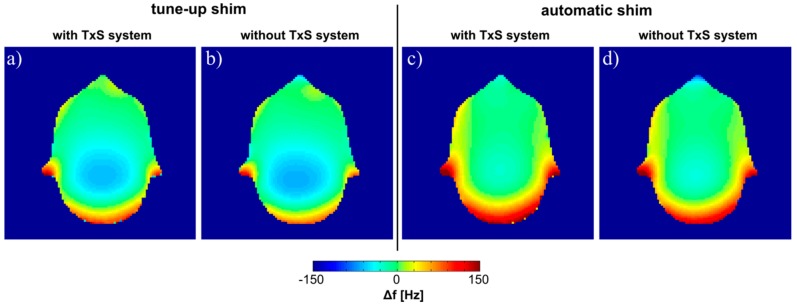
Influence of the liquid drive on B_0_. Field maps with and without the TxS system for the tune-up shim (**a**,**b**) and after applying the automatic shimming procedure of the scanner (**c**,**d**).

Static field maps acquired with the body coil with and without the TxS system for two different shim configurations are shown in Figure 6. It is demonstrated that the influence of the complete TxS system on the static magnetic field of the scanner is negligible. In particular, no influence on the achievable shimming performance is expected. This holds true for imaging with a large FOV, as well as for local shimming used in single-voxel spectroscopy, for instance. Results evaluating the B_0_ inhomogeneity in terms of the standard deviation of frequency values calculated for both, the entire phantom, and the slice shown in Figure 6 are summarized in Table 1 for the two shimming configurations investigated.

### 3.4. Performance Comparison with Commercial 16-Channel Coil

**Figure 7:**
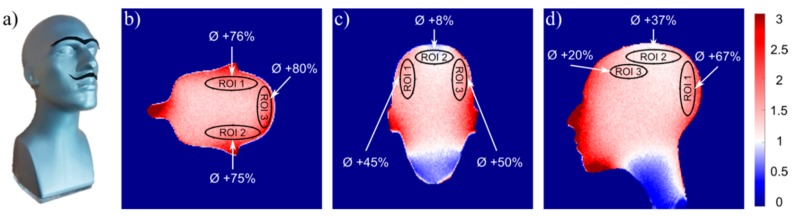
SNR gain of the 24-channel PET/MR coil array versus the 16-channel commercial reference coil. (**a**) Photograph of the head-shaped gel phantom used for measurements, (**b**) central coronal slice, (**c**) central sagittal slice, and (**d**) central transversal slice. The mean relative SNR gain (red) is indicated for each region-of-interest (ROI). Losses are shown in blue.

Maps of the SNR gain achieved with the developed coil versus the reference coil are depicted in Figure 7. Results are shown for the sagittal, transversal, and coronal slices through the center of the phantom. The new RF coil offers an SNR gain especially in transversal slices through the brain, where the SNR for cortical regions is improved on average by 66% to 80%. For sagittal and coronal slices in cortical regions, our RF coil offers an SNR improvement of 20% to 67% and 8% to 50% in mean SNR, respectively. In contrast, an SNR decrease is observed for the anterior part of the neck. This is because the commercial reference coil has a split-type design enabling the placement of coil elements closer to this body part, which results in a higher SNR in this region.

### 3.5. Parallel Imaging Performance: g-Factor Maps

**Figure 8:**
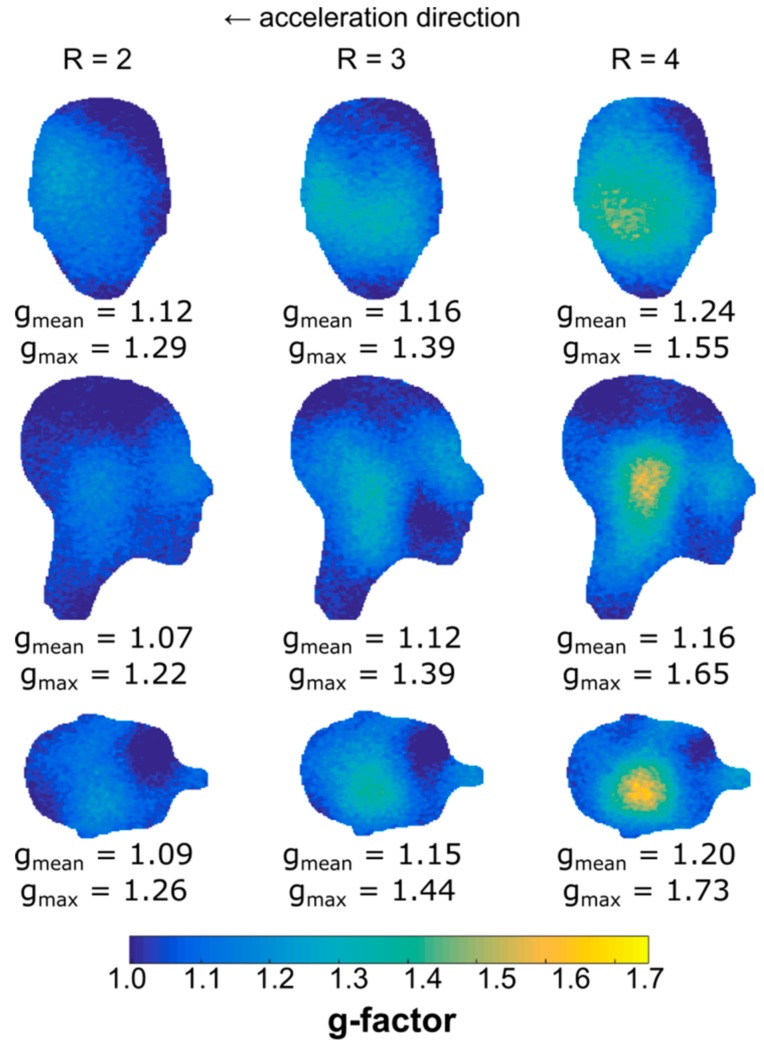
g-factor maps. From top to bottom: coronal, sagittal, and transversal slices through the center of the gel phantom shown in Figure 7 for acceleration factors *R* = 2, 3, and 4 (left to right).

The g-factor maps in coronal, sagittal, and transversal slices through the center of the phantom for acceleration factors *R* = 2, 3, and 4 are depicted in Figure 8. The acceleration direction was left to right for the coronal slice, and anterior to posterior for the sagittal and transversal slices. Mean and maximum g-factors are given below each slice, where the highest observed value was 1.73 (transversal slice, *R* = 4).

### 3.6. In Vivo MRI

**Figure 9:**
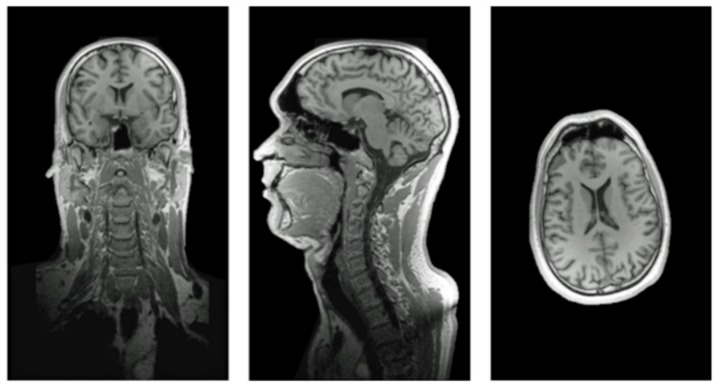
In vivo MR images. First in vivo measurements (MPRAGE images) using the complete device (i.e., RF coil integrated with TxS system).

First in vivo MR measurements with the developed head and neck coil were successfully completed. An adequate coverage of the male volunteer’s head and neck is achieved. Coronal, sagittal, and transversal images are shown in Figure 9.

### 3.7. PET Performance: Attenuation Correction Calculation

**Figure 10:**
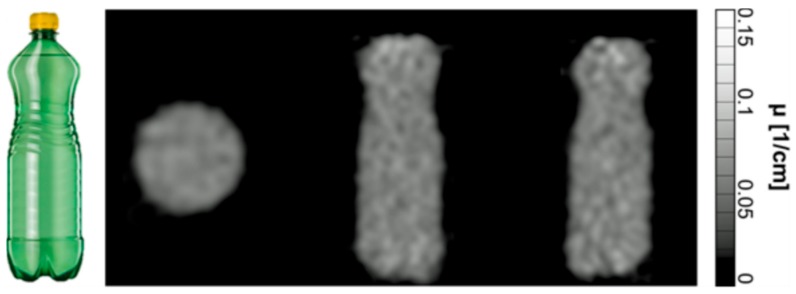
PET attenuation map. **Left**: Water bottle (1 L) used for the first PET AC calculations. **Right**: Reconstructed attenuation maps in all three directions using acquired data from the transmission scan.

A photograph and the reconstructed attenuation maps of the water bottle are shown in Figure 10. The mean calculated linear attenuation coefficient µ was (0.093 ± 0.015) cm^−1^ for the whole bottle. This value is in very good agreement with the literature value [39] of 0.096 cm^−1^.

## 4. Discussion

A PET/MR receive-only coil array integrated with a transmission source system for accurate AC map calculation for head and neck PET/MR applications is presented.

By using thin copper wire for the coil elements and placing the preamplifiers outside the PET FOV, as well as careful housing design, PET attenuation due to the RF coil itself was minimized. This was validated by Monte-Carlo simulations yielding a global scatter-fraction of 16.4% for the proposed head coil system (i.e., TxS system plus RF coil array) and the head phantom, which is well below the reported scatter-fraction for whole body 3D imaging of 30–50% [10]. These results confirm the viability of our design to acquire highly sensitive PET data that can be reconstructed using standard algorithms. In addition, the photon count loss of 26% of the whole head coil system is comparable to the reported 17% loss of a state-of-the-art PET-compatible RF coil [6].

The impact of the developed device, i.e., RF coil and TxS system, on the RF transmit field and the static magnetic field was found to be marginal. This indicates that MR measurements will not be compromised due to the presence of the developed additional hardware.

Further, it has been demonstrated that the developed RF coil outperforms a commercial 16-channel head and neck coil in terms of SNR and parallel imaging performance in most regions of a head and neck phantom. Regions with lower SNR than provided by the 16-channel coil are the anterior part of the neck and the top part of the head. In the case of the neck, the presented PET/MR coil was built on a fixed ellipsoid where the two elements positioned to cover the anterior part of the neck, are further away from the target region than for the reference coil. This is due to the fact that our RF coil is not designed to be split into an anterior and a posterior part. Therefore, the coil elements cannot be positioned closer to the neck since the whole head has to pass through the opening. On the top part of the head, the presented coil layout does not include an element covering this region directly.

First attenuation correction results obtained from transmission scans with the new hardware are in excellent agreement with literature values and, therefore, serve as proof of concept for the presented AC method. The shape of the phantom bottle could be accurately reconstructed despite the low activity and short scan time used. For the very first transmission scans presented in this work, a low activity of 14 MBq was used; the quality of the calculated attenuation maps increases significantly when using a typical activity of 100 MBq. In a PET specific study, the performance of the novel device was investigated with more complex phantoms [26]. These phantoms included cylindrical inserts of polytetrafluoroethylene (PTFE) and air which could be reconstructed with the true geometrical dimensions. However, the linear attenuation coefficient of PTFE was underestimated, which needs further investigation. Future work will compare the device to state-of-the-art methods for attenuation correction in PET/MR experiments in an animal study.

The novel, fully-integrated hardware enables transmission measurements at 511 keV, which distinguishes the method from CT, the “silver standard” for attenuation coefficient mapping. With the proposed method attenuation coefficients—including those of the RF coil and the patient—can be measured at the appropriate energy of 511 keV. The technique yields accurate AC results for non-TOF systems and may potentially lead to more accurate tracer quantification than methods based on segmentation of the MR data currently used in commercial systems. In addition, it can serve as a true gold standard for the measurement of attenuation coefficients and be used for the validation of MR-image based methods.

The developed device could be applied in head and neck cancer imaging, especially for scenarios where PET/MR is expected to bring an advantage over PET/CT, including the perineural spread of tumors and the infiltration of important anatomical landmarks, such as the prevertebral fascia and great vessel walls [40,41].

## Figures and Tables

**Table 1:** B_0_ homogeneity. Standard deviation values of the frequency, measured with and without the TxS system for tune-up shim and automatic shim. Data were evaluated either for the entire phantom or only for the slice shown in Figure 6.

		Standard Deviation of ∆*f* (Hz)	
		with TxS System	without TxS System
tune-up shim	phantom	97.0	97.5
	slice	40.4	36.8
automatic shim	phantom	72.5	75.9
	slice	45.4	43.0
